# Radioprotectant Activity of Dicopper(II) Tetrakis(3,5- Diisopropylsalicylate) and Manganese(II) bis(3,5- Diisopropylsalicylate) Alone and in Combination

**DOI:** 10.1155/MBD.1999.127

**Published:** 1999

**Authors:** John A. Kuykendall, III, Robert H. Ebert, II, lbrahim H. El-Sayed, Paula K. Roberson, Van Anh Pham-Tran, Billynda L. Booth, Tiffany Brooks, William M. Willingham, John R. J. Sorenson

**Affiliations:** 1 University of Arkansas for Medical Sciences Campus Division of Medicinal Chemistry College of Pharmacy Little Rock Arkansas 72205 USA; 2 University of Arkansas for Medical Sciences Campus Arkansas Cancer Research Center Little Rock Arkansas 72205 USA; 3 Research Center University of Arkansas at Pine Bluff Pine Bluff Arkansas 71601 USA

## Abstract

Dicopper(ll) tetrakis(3,5-diisopropylsalicylate), (Cu(II)_2_(3,5-DIPS)_4_, manganese(II) bis(3,5-diisopropylsalicylate), Mn(II)3,5-DIPS)_2_ or combinations of them were used to treat gamma-irradIated mice in examining the possibility that combination treatments might be more effective in increasing survival than treatment with either complex alone. Doses of 0, 10, 20, or 40 μmol of each complex per kilogram of body mass were administered subcutaneously in a factorial design before 9 Gy gamna irradiation, an LD_90_ dose of irradiation. Doses of 0, 10, 20, or 40 μmol Cu(II)_2_(3, 5-DIPS)_4_ per kg of body mass produced 12, 28, 28, or 36 % survival, respectively, while doses of 0, 10, 20, or 40 μmol (II)_3_, 5-DIPS)_2_ per kg of body mass prduced 12, 36, 20, or 24 % survival, respectively. However, the combination of 20 μmol Cu(II)_2_(3, 5-DIPS)_4_ and 10 μmol Mn(II)(3, 5−DIPS)_2_ produced the greatest survival, 48 %, which was 300 % greater than vehicle-treated mice (P=0.01). It is concluded that specific combination treatments can be used to maximize survival of lethally irradiated mice.


### RADIOPROTECTANT ACTIVITY OF DICOPPER(II) TETRAKIS(3,5-DIISOPROPYLSALICYLATE) AND MANGANESE(II) BIS(3,5-DIISOPROPYLSALICYLATE) ALONE AND IN COMBINATION

John A. Kuykendall, III[2], Robert H. Ebert, II[1a], Ibrahim H. El-Sayed[1a], Paula K. Roberson[1b], Van Anh Pham-Tran[1a], Billynda L. Booth[1a], Tiffany Brooks[1a], William M. Willingham[2], and John R. J. Sorenson*[1a]

[1] University of Arkansas for Medical Sciences Campus, Little Rock, Arkansas 72205, USA
[a] Division of Medicinal Chemistry, College of Pharmacy,
e-mail: sorensonjohnr@exchange.uams.edu
[b] Arkansas Cancer Research Center
[2] Research Center, University of Arkansas at Pine Bluff, Pine Bluff, Arkansas 71601, USA

Abstract

Dicopper(II) tetrakis(3,5-diisopropylsalicylate), $(Cu(II)_2(3,5\text{-}DIPS)_4$, manganese(II) bis(3,5-diisopropylsalicylate), $Mn(II)(3,5\text{-}DIPS)_2$ or combinations of them were used to treat gamma-irradiated mice in examining the possibility that combination treatments might be more effective in increasing survival than treatment with either complex alone. Doses of 0, 10, 20, or 40 µmol of each complex per kilogram of body mass were administered subcutaneously in a factorial design before 9 Gy gamma irradiation, an $LD_{90}$ dose of irradiation. Doses of 0, 10, 20, or 40 µmol $Cu(II)_2(3,5\text{-}DIPS)_4$ per kg of body mass produced 12, 28, 28, or 36 % survival, respectively, while doses of 0, 10, 20, or 40 µmol $Mn(II)(3,5\text{-}DIPS)_2$ per kg of body mass produced 12, 36, 20, or 24 % survival, respectively. However, the combination of 20 µmol $Cu(II)_2(3,5\text{-}DIPS)_4$ and 10 µmol $Mn(II)(3,5\text{-}DIPS)_2$ produced the greatest survival, 48 %, which was 300 % greater than vehicle-treated mice (P=0.01). It is concluded that specific combination treatments can be used to maximize survival of lethally irradiated mice.

### Introduction

Both $Cu(II)_2(3,5\text{-}DIPS)_4$ and $Mn(II)(3,5\text{-}DIPS)_2$ have been found to be effective radioprotectants in $LD_{50/30}$ and $LD_{100/30}$ radiation paradigms [1 and references therein]. Other essential metalloelement compounds including chelates of zinc and iron have also been found to have radioprotectant activity. It is suggested that the radioprotectant and radiorecovery activity of various essential metalloelement compounds is based upon roles of these essential metalloelement-dependent enzymes in repair of radiation injury [1].

Since essential metalloelement-dependent enzymes require a specific metalloelement(s) for optimal activity, treatment with a single metalloelement will facilitate the role of that class of metalloelement-dependent enzymes in responding to radiation injury and increasing survival. A priori the response to overcome radiation injury by facilitating a single metalloelement class of enzymes is limited. However, facilitating the response to radiation injury by enhancing the activity of more than one class of essential metalloelement-dependent enzymes should increase the effectiveness of treating radiation injury and increase survival.

In using either a single or multiple essential metalloelement treatment approach to overcoming radiation injury there is plausible risk with regard to the incorporation of an essential metalloelement into the wrong apoenzyme to yield a less active or inactive metalloelement-dependent enzyme. Consequently, maximal effectiveness of this approach to treatment may depend upon the use of a somewhat narrow range of combination doses.

We are reporting the radioprotectant activity (survival) of lethally irradiated mice treated with $Cu(II)_2(3,5\text{-}DIPS)_4$, $Mn(II)(3,5\text{-}DIPS)_2$, or combinations of these complexes. The 900 Gy dose selected for these studies was chosen to increase radiation injury as a more rigorous evaluation of combination treatments in providing radioprotectant activity.

### Materials and Methods

The synthesis $Cu(II)_2(3,5\text{-}DIPS)_4$ and $Mn(II)(3,5\text{-}DIPS)_2$ have been described in detail [2]. Both compounds were first wetted with a volume of Propylene Glycol (Sigma) sufficient to yield a final solution or suspension containing 4 % Propylene Glycol in 1.4 % of Polyvinyl

Alcohol (Sigma # 1763) in Saline (Travenol). Incomplete vehicle, 1.4 % Polyvinyl Alcohol in Saline, was prepared by dissolving 1.4g of Polyvinyl Alcohol in stirred Saline heated at 90°C for one hour. Complete vehicle was prepared by adding 4 ml of Propylene Glycol to 96 ml of incomplete vehicle.

Three hundred and twenty µmol (341.1 mg) of $Cu(II)_2(3,5\text{-DIPS})_4(H_2O)_3$, Mr = 1,066 Daltons, was wetted with 4 ml of Propylene Glycol in a Potter-Elvehjem homogenizer while cooling in an ice bath to prevent the increase in viscosity associated with homogenization without cooling. The homogenate was then transferred to a 200 ml graduated cylinder with sufficient incomplete vehicle to make 100 ml. The cylinder was covered with Parafilm (American National Can) and vortex stirred to insure homogeneity. This suspension containing 1.6 µmol $Cu(II)_2(3,5\text{-DIPS})_4$ per 0.5 ml was vortex stirred before all subsequent usage.

Fifty ml of the 1.6 µmol/0.5 ml stirred suspension was diluted with 50 ml of complete vehicle and the diluted 100 ml suspension vortex stirred. This suspension contained 0.8 µmol $Cu(II)_2(3,5\text{-DIPS})_4$ per 0.5 ml. Treatment of a 20 g mouse with 0.5 ml of this suspension provides 40 µmol/kg of body mass.

Fifty ml of the 0.8 µmol/0.5 ml stirred suspension was diluted with 50 ml of complete vehicle and the diluted 100 ml suspension vortex stirred. This suspension contained 0.4 µmol $Cu(II)_2(3,5\text{-DIPS})_4$ per 0.5 ml. Treatment of a 20 g mouse with 0.5 ml of this suspension provides 20 µmol/kg of body mass.

Fifty ml of the 0.4 µmol/0.5 ml stirred suspension was diluted with 50 ml of complete vehicle and the diluted 100 ml suspension vortex stirred. This suspension contained 0.2 µmol $Cu(II)_2(3,5\text{-DIPS})_4$ per 0.5 ml. Treatment of a 20 g mouse with 0.5 ml of this suspension provides 10 µmol/kg of body mass.

Three hundred and twenty µmol (168.3 mg) of $Mn(II)(3,5\text{-DIPS})_2(H_2O)_{1.5}$, Mr = 526 Daltons, was wetted with 4 ml of Propylene Glycol in a Potter-Elvehjem homogenizer while cooling in an ice bath to prevent the increase in viscosity associated with homogenization without cooling. The homogenate was then transferred to a 200 ml graduated cylinder with sufficient incomplete vehicle to make 100 ml. The cylinder was covered with Parafilm (American National Can) and vortex stirred to insure homogeneity of what appeared to be a true solution. This apparent solution was still vortex stirred to guarantee homogeneity before all subsequent dilutions. This solution contained 1.6 µmol $Mn(II)(3,5\text{-DIPS})_2$ per 0.5 ml.

Fifty ml of the 1.6 µmol/0.5 ml stirred solution was diluted with 50 ml of complete vehicle and the diluted 100 ml solution vortex stirred. This solution contained 0.8 µmol $Mn(II)(3,5\text{-DIPS})_2$ per 0.5 ml. Treatment of a 20 g mouse with 0.5 ml of this suspension provides 40 µmol/kg of body mass.

Fifty ml of the 0.8 µmol/0.5 ml stirred solution was diluted with 50 ml of complete vehicle and the diluted 100 ml solution vortex stirred. This solution contained 0.4 µmol $Mn(II)(3,5\text{-DIPS})_2$ per 0.5 ml. Treatment of a 20 g mouse with 0.5 ml of this suspension provides 20 µmol/kg of body mass.

Fifty ml of the 0.4 µmol/0.5 ml stirred solution was diluted with 50 ml of complete vehicle and the diluted 100 ml solution vortex stirred. This solution contained 0.2 µmol $Mn(II)(3,5\text{-DIPS})_2$ per 0.5 ml. Treatment of a 20 g mouse with 0.5 ml of this suspension provides 10 µmol/kg of body mass.

Ten ml of 0.4 µmol $Cu(II)_2(3,5\text{-DIPS})_4$ suspension per 0.5 ml vortex stirred with 10 ml of complete vehicle gave a 20 ml suspension containing 0.2 µmol $Cu(II)_2(3,5\text{-DIPS})_4$ per 0.5 ml. Treatment of a 20 g mouse with 0.5 ml of this suspension provides 10 µmol $Cu(II)_2(3,5\text{-DIPS})_4$ /kg of body mass.

Ten ml of 0.8 µmol $Cu(II)_2(3,5\text{-DIPS})_4$ suspension per 0.5 ml vortex stirred with 10 ml of complete vehicle gave a 20 ml suspension containing 0.4 µmol $Cu(II)_2(3,5\text{-DIPS})_4$ per 0.5 ml. Treatment of a 20 g mouse with 0.5 ml of this suspension provides 20 µmol $Cu(II)_2(3,5\text{-DIPS})_4$ /kg of body mass.

Ten ml of 1.6 µmol $Cu(II)_2(3,5\text{-DIPS})_4$ suspension per 0.5 ml vortex stirred with 10 ml of complete vehicle gave a 20 ml suspension containing 0.8 µmol $Cu(II)_2(3,5\text{-DIPS})_4$ per 0.5 ml. Treatment of a 20 g mouse with 0.5 ml of this suspension provides 40 µmol $Cu(II)_2(3,5\text{-DIPS})_4$ /kg of body mass.

Ten ml of 0.4 µmol $Mn(II)(3,5\text{-DIPS})_2$ suspension per 0.5 ml vortex stirred with 10 ml of complete vehicle gave a 20 ml suspension containing 0.2 µmol $Mn(II)(3,5\text{-DIPS})_2$ per 0.5 ml. Treatment of a 20 g mouse with 0.5 ml of this suspension provides 10 µmol $Mn(II)(3,5\text{-DIPS})_2$ /kg of body mass.

Ten ml of 0.8 µmol $Mn(II)(3,5\text{-DIPS})_2$ suspension per 0.5 ml vortex stirred with 10 ml of complete vehicle gave a 20 ml suspension containing 0.4 µmol $Mn(II)(3,5\text{-DIPS})_2$ per 0.5

ml. Treatment of a 20 g mouse with 0.5 ml of this suspension provides 20 μmol Mn(II)(3,5-DIPS)$_2$ /kg of body mass.

Ten ml of 1.6 μmol Mn(II)(3,5-DIPS)$_2$ suspension per 0.5 ml vortex stirred with 10 ml of complete vehicle gave a 20 ml suspension containing 0.8 μmol Mn(II)(3,5-DIPS)$_2$ per 0.5 ml. Treatment of a 20 g mouse with 0.5 ml of this suspension provides 40 μmol Mn(II)(3,5-DIPS)$_2$ /kg of body mass.

Ten ml of 0.4 μmol Cu(II)$_2$(3,5-DIPS)$_4$ suspension per 0.5 ml vortex stirred with 10 ml of 0.4 μmol Mn(II)(3,5-DIPS)$_2$ suspension per 0.5 ml gave a 20 ml suspension containing 0.2 μmol Cu(II)$_2$(3,5-DIPS)$_4$ and Mn(II)(3,5-DIPS)$_2$ per 0.5 ml. Treatment of a 20 g mouse with 0.5 ml of this suspension provides 10 μmol Cu(II)$_2$(3,5-DIPS)$_4$ and Mn(II)(3,5-DIPS)$_2$ /kg of body mass.

Ten ml of 0.8 μmol Cu(II)$_2$(3,5-DIPS)$_4$ suspension per 0.5 ml vortex stirred with 10 ml of 0.4 μmol Mn(II)(3,5-DIPS)$_2$ suspension per 0.5 ml gave a 20 ml suspension containing 0.4 μmol Cu(II)$_2$(3,5-DIPS)$_4$ and 0.2 μmol Mn(II)(3,5-DIPS)$_2$ per 0.5 ml. Treatment of a 20 g mouse with 0.5 ml of this suspension provides 20 μmol Cu(II)$_2$(3,5-DIPS)$_4$ and 10 μmol Mn(II)(3,5-DIPS)$_2$ /kg of body mass.

Ten ml of 1.6 μmol Cu(II)$_2$(3,5-DIPS)$_4$ suspension per 0.5 ml vortex stirred with 10 ml of 0.4 μmol Mn(II)(3,5-DIPS)$_2$ suspension per 0.5 ml gave a 20 ml suspension containing 0.8 μmol Cu(II)$_2$(3,5-DIPS)$_4$ and 0.2 μmol Mn(II)(3,5-DIPS)$_2$ per 0.5 ml. Treatment of a 20 g mouse with 0.5 ml of this suspension provides 40 μmol Cu(II)$_2$(3,5-DIPS)$_4$ and 10 μmol Mn(II)(3,5-DIPS)$_2$ /kg of body mass.

Ten ml of 0.4 μmol Cu(II)$_2$(3,5-DIPS)$_4$ suspension per 0.5 ml vortex stirred with 10 ml of 0.8 μmol Mn(II)(3,5-DIPS)$_2$ suspension per 0.5 ml gave a 20 ml suspension containing 0.2 μmol Cu(II)$_2$(3,5-DIPS)$_4$ and 0.4 μmol Mn(II)(3,5-DIPS)$_2$ per 0.5 ml. Treatment of a 20 g mouse with 0.5 ml of this suspension provides 10 μmol Cu(II)$_2$(3,5-DIPS)$_4$ and 20 μmol Mn(II)(3,5-DIPS)$_2$ /kg of body mass.

Ten ml of 0.8 μmol Cu(II)$_2$(3,5-DIPS)$_4$ suspension per 0.5 ml vortex stirred with 10 ml of 0.8 μmol Mn(II)(3,5-DIPS)$_2$ suspension per 0.5 ml gave a 20 ml suspension containing 0.4 μmol Cu(II)$_2$(3,5-DIPS)$_4$ and 0.4 μmol Mn(II)(3,5-DIPS)$_2$ per 0.5 ml. Treatment of a 20 g mouse with 0.5 ml of this suspension provides 20 μmol Cu(II)$_2$(3,5-DIPS)$_4$ and Mn(II)(3,5-DIPS)$_2$ /kg of body mass.

Ten ml of 1.6 μmol Cu(II)$_2$(3,5-DIPS)$_4$ suspension per 0.5 ml vortex stirred with 10 ml of 0.8 μmol Mn(II)(3,5-DIPS)$_2$ suspension per 0.5 ml gave a 20 ml suspension containing 0.8 μmol Cu(II)$_2$(3,5-DIPS)$_4$ and 0.4 μmol Mn(II)(3,5-DIPS)$_2$ per 0.5 ml. Treatment of a 20 g mouse with 0.5 ml of this suspension provides 40 μmol Cu(II)$_2$(3,5-DIPS)$_4$ and 20 μmol Mn(II)(3,5-DIPS)$_2$ /kg of body mass.

Ten ml of 0.4 μmol Cu(II)$_2$(3,5-DIPS)$_4$ suspension per 0.5 ml vortex stirred with 10 ml of 1.6 μmol Mn(II)(3,5-DIPS)$_2$ suspension per 0.5 ml gave a 20 ml suspension containing 0.2 μmol Cu(II)$_2$(3,5-DIPS)$_4$ and 0.8 μmol Mn(II)(3,5-DIPS)$_2$ per 0.5 ml. Treatment of a 20 g mouse with 0.5 ml of this suspension provides 10 μmol Cu(II)$_2$(3,5-DIPS)$_4$ and 40 μmol Mn(II)(3,5-DIPS)$_2$ /kg of body mass.

Ten ml of 0.8 μmol Cu(II)$_2$(3,5-DIPS)$_4$ suspension per 0.5 ml vortex stirred with 10 ml of 1.6 μmol Mn(II)(3,5-DIPS)$_2$ suspension per 0.5 ml gave a 20 ml suspension containing 0.4 μmol Cu(II)$_2$(3,5-DIPS)$_4$ and 0.8 μmol Mn(II)(3,5-DIPS)$_2$ per 0.5 ml. Treatment of a 20 g mouse with 0.5 ml of this suspension provides 20 μmol Cu(II)$_2$(3,5-DIPS)$_4$ and 40 μmol Mn(II)(3,5-DIPS)$_2$ /kg of body mass.

Ten ml of 1.6 μmol Cu(II)$_2$(3,5-DIPS)$_4$ suspension per 0.5 ml vortex stirred with 10 ml of 1.6 μmol Mn(II)(3,5-DIPS)$_2$ suspension per 0.5 ml gave a 20 ml suspension containing 0.8 μmol Cu(II)$_2$(3,5-DIPS)$_4$ and Mn(II)(3,5-DIPS)$_2$ per 0.5 ml. Treatment of a 20 g mouse with 0.5 ml of this suspension provides 40 μmol Cu(II)$_2$(3,5-DIPS)$_4$ and Mn(II)(3,5-DIPS)$_2$ /kg of body mass.

These solutions were used to provide factorial design treatments shown in Table I.

Sixteen groups of 25 randomized 10 week old, young adult, 20 to 22 g female C57BL/6 mice housed 5 per cage, and fed mouse chow pellets and water ad libitum, with 12 hr light (6:00 am to 6:00 pm) and dark cycles for 3 weeks prior to treatment. Each group of 25 mice was treated subcutaneously in the dorsal nape of the neck beginning at 9:00 am with one of the sixteen treatments: either vehicle or one of the single or combination treatments, three to eight hours before gamma-irradiation (900 cGy, 124.5 cGy/min) in a Shepard Mark $^{137}$Cs irradiator beginning at 12:00 pm. All 25 mice in each treatment group were irradiated over a 29 minute period and survival determined over a 30 day post-treatment and irradiation period. The 900 cGy dose was selected to increase radiation injury beyond the $LD_{50/30}$ dose of 800 c Gy (1) to more rigorously examine these combination therapies for radioprotectant activity.

Statistical comparisons of the proportion surviving among treatment groups were made using Fisher's Exact Test. The software used was StatXact -3 (Cytel Software Corporation).

All animal experiments were approved by the Institute's Animal Care and Use Committee which has an Animal Welfare Assurance on file with the Office of Protection from Research Risks.

### Results

As shown in Table I, treatment with all single and nearly all combination doses of $Cu(II)_2(3,5\text{-}DIPS)_4$ and/or $Mn(II)(3,5\text{-}DIPS)_2$, were effective in increasing survival. Treatment with 10 μmol, 20 μmol, or 40 μmol $Cu(II)_2(3,5\text{-}DIPS)_4$ gave survivals of 28 %, 28 %, or 36 %, respectively, as shown in Figure 1, which represent 133 %, 133 %, or 200 % increases in survival compared vehicle-treated mice (Table II).

Table I. Percent survival of treated mice.

| Micromole $Mn(II)(3,5\text{-}DIPS)_2$ / kg of body mass | Micromole $Cu(II)_2(3,5\text{-}DIPS)_4$ / kg of body mass. | | | |
|---|---|---|---|---|
| | 0 | 10 | 20 | 40 |
| 0 | 12[a] | 28[a] | 28[a] | 36[a,b] |
| 10 | 36[a,b] | 36[a,b] | 48[b,v] | 36[a,b] |
| 20 | 20[a] | 32[a] | 8[a] | 36[a,b] |
| 40 | 24[a] | 20[a] | 20[a] | 24[a] |

[a]: denotes a lack of statistically significant difference compared to the vehicle-treated group.
[b]: denotes a statistically significant difference from the 20 μmol $Cu(II)_2(3,5\text{-}DIPS)_4$ and 20 μmol $Mn(II)(3,5DIPS)_2$-treated group($p < 0.05$), Fisher's Exact Test.
[c]: denotes a statistically significant difference compared to the vehicle-treated group ($p = 0.01$), Fisher's Exact Test.

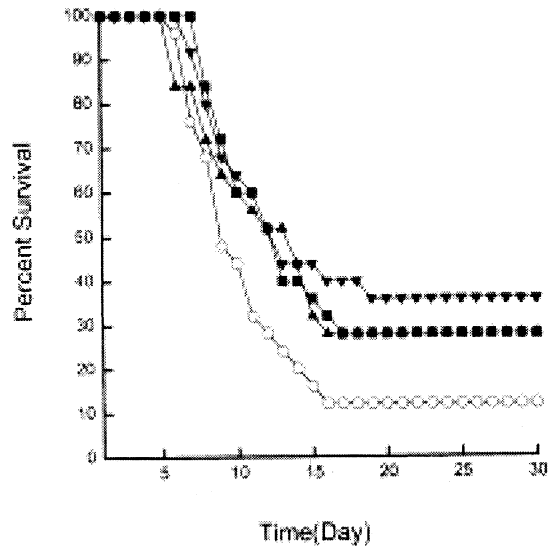

Figure 1. Radioprotectant activity of vehicle (○), 10 μmol (■), 20 μmol (▲), or 40 μmol (▼) $Cu(II)_2(3,5\text{-}DIPS)_4$ per kg of body mass in 900 cGy irradiated mice.

Treatment with 10 μmol, 20 μmol, or 40 μmol $Mn(II)(3,5\text{-}DIPS)_2$ gave survivals of 36%, 20%, 24%, respectively, as shown in Figure 2, which represent 200%, 167%, or 100% increases in survival compared to vehicle-treated mice (Table II).

Table II. Percent increase in survival of complex-treated mice.

| Micromole $Mn(II)(3,5\text{-}DIPS)_2$ / kg of body mass | Micromole $Cu(II)_2(3,5\text{-}DIPS)_4$ / kg of body mass. | | | |
|---|---|---|---|---|
| | 0 | 10 | 20 | 40 |
| 0 | | 133 | 133 | 200 |
| 10 | 200 | 200 | 300 | 200 |
| 20 | 67 | 167 | -33 | 200 |
| 40 | 100 | 67 | 67 | 100 |

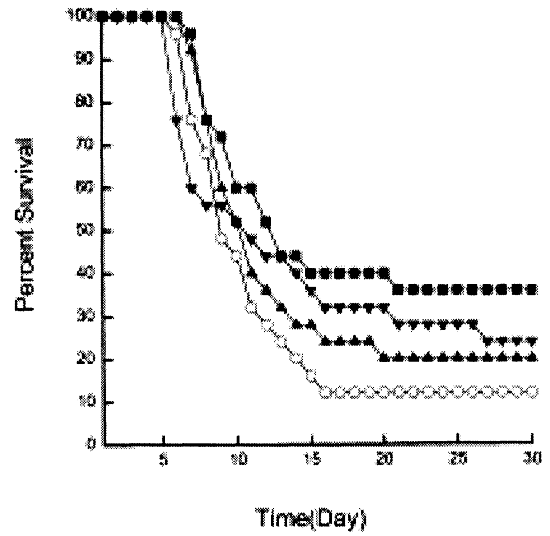

Figure 2. Radioprotectant activity of vehicle (○), 10 μmol (■), 20 μmol (▲), or 40 μmol (▼) $Mn(II)(3,5\text{-}DIPS)_2$ per kg of body mass in 900 cGy irradiated mice.

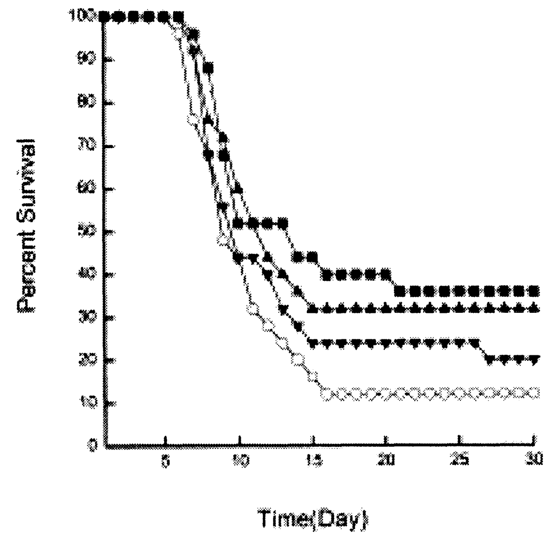

Figure 3. Radioprotectant activity of vehicle (○), 10 μmol $Cu(II)_2(3,5\text{-}DIPS)_4$ and 10 μmol $Mn(II)(3,5\text{-}DIPS)_2$ (■), 10 μmol $Cu(II)_2(3,5\text{-}DIPS)_4$ and 20 μmol $Mn(II)(3,5\text{-}DIPS)_2$ (▲), or 10 μmol $Cu(II)_2(3,5\text{-}DIPS)_4$ and 40 μmol $Mn(II)(3,5\text{-}DIPS)_2$ (▼) per kg of body mass in 900 cGy irradiated mice.

Nearly all treatments with combinations of $Cu(II)_2(3,5\text{-}DIPS)_4$ and $Mn(II)(3,5\text{-}DIPS)_2$ increased survival as shown in Figures 3, 4, and 5 with the exception of the 20 μmol

$Cu(II)_2(3,5\text{-}DIPS)_4$ and $Mn(II)(3,5\text{-}DIPS)_2$ /kg of body mass treatment shown in Figure 4. These data show that addition of 10 μmol $Mn(II)(3,5\text{-}DIPS)_2$ to 10 μmol or 20 μmol $Cu(II)_2(3,5\text{-}DIPS)_4$ increased survival. The combination of 20 μmol $Cu(II)_2(3,5\text{-}DIPS)_4$ and 10 μmol $Mn(II)(3,5\text{-}DIPS)_2$ /kg of body mass was most effective in increasing survival. However, increasing the dose of $Mn(II)(3,5\text{-}DIPS)_2$ beyond 10 μmol/kg of body mass seems to have decreased survival as shown in Tables I and II and the combination of 20 μmol $Cu(II)_2(3,5\text{-}DIPS)_4$ and 20 μmol $Mn(II)(3,5\text{-}DIPS)_2$ was ineffective in increasing survival.

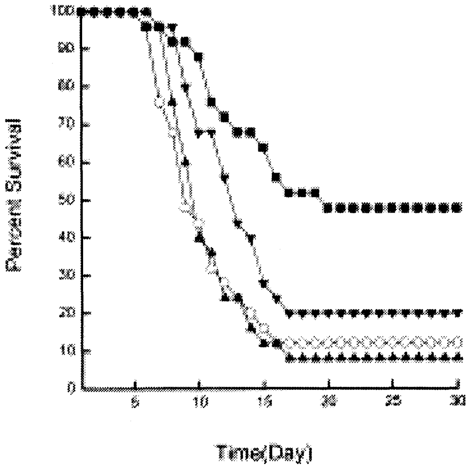

Figure 4. Radioprotectant activity of vehicle (O), 20 μmol $Cu(II)_2(3,5\text{-}DIPS)_4$ and 10 μmol $Mn(II)(3,5\text{-}DIPS)_2$ (■), 20 μmol $Cu(II)_2(3,5\text{-}DIPS)_4$ and 20 μmol $Mn(II)(3,5\text{-}DIPS)_2$ (▲), or 20 μmol $Cu(II)_2(3,5\text{-}DIPS)_4$ and 40 μmol $Mn(II)(3,5\text{-}DIPS)_2$ (▼) per kg of body mass in 900 cGy irradiated mice.

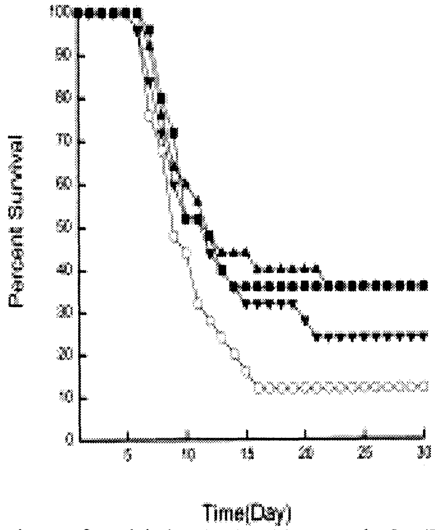

Figure 5. Radioprotectant activity of vehicle (O), 40 μmol $Cu(II)_2(3,5\text{-}DIPS)_4$ and 10 μmol $Mn(II)(3,5\text{-}DIPS)_2$ (■), 40 μmol $Cu(II)_2(3,5\text{-}DIPS)_4$ and 20 μmol $Mn(II)(3,5\text{-}DIPS)_2$ (▲), or 40 μmol $Cu(II)_2(3,5\text{-}DIPS)_4$ and 40 μmol $Mn(II)(3,5\text{-}DIPS)_2$ (▼) per kg of body mass in 900 cGy irradiated mice.

Discussion

None of the single or combination doses selected for this study caused acute toxicity, death in a treatment group prior to death in the vehicle-treated group. As shown in Figures 1 to 5, there were no deaths in any group prior to day 5 after treatment.

Data presented in Tables I and II and Figure 1 show that treatment with $Cu(II)_2$(3,5-DIPS)$_4$ caused an increase in survival in 900 cGy irradiated mice as the dose was increased from 10 μmol/kg to 40 μmol/kg with no change in survival when the dose was increased from 10 μmol/kg to 20 μmol/kg. Treatment with 10 μmol Mn(II)(3,5-DIPS)$_2$/kg caused an increase in survival compared with vehicle-treated mice. However, treatment with the higher doses, 20 μmol and 40 μmol/kg, did not cause a dose-related increase in survival which decreased with increasing dose of Mn(II)(3,5-DIPS)$_2$ above 10 μmol/kg (Tables I and II and Figures 2, 3, 4, and 5). This decrease in survival may be due to a Mn-induced interference wherein Mn may be inappropriately incorporated into an apoenzyme that requires some other essential metalloelement to maximally fulfill its role in repair of a 900 cGy radiation injury. This possible interference, which may arise with larger doses of irradiation, is offered as a rationale in explaining why Mn(II)(3,5-DIPS)$_2$ given to 800 cGy irradiated mice gave 100 % survival [3]. The greater radiation injury caused by 900 cGy may have caused an increased essential metalloelement-dependent response wherein there is an increased physiological response involving other essential metalloelement-dependent enzymes that may be less active as a result of the inappropriate incorporation of Mn.

This decrease in survival associated with increasing dose of Mn(II)(3,5-DIPS)$_2$ from 10 μmol to 20 μmol, or 40 μmol/kg was also observed for all groups treated with $Cu(II)_2$(3,5-DIPS)$_4$ and Mn(II)(3,5-DIPS)$_2$ although this decrease was less severe when combination treatment involved treatments with 40 μmol $Cu(II)_2$(3,5-DIPS)$_4$/kg of body mass.

Groups of mice treated with 20 μmol $Cu(II)_2$(3,5-DIPS)$_4$ and 20 μmol Mn(II)(3,5-DIPS)$_2$ had the lowest survival. Early in the course of this experiment, all mice in one cage died on a single day following the determination of survival. Death of all mice in a single cage is unusual in our experience, but may have been due to cannibalization of a mouse, that had died as a result of pathogenic infection associated with irradiation-induced immunoincompetence, by mice that were also immunoincompetent. If only one mouse would have died in this group as a result of infection due to radiation-induced immunoincompetence and there were no other deaths, survival for this group would have been 32 %, which is still less than the 48 % survival for the group treated with 20 μmol $Cu(II)_2$(3,5-DIPS)$_4$ and 10 μmol Mn(II)(3,5-DIPS)$_2$/kg of body mass, and the consistent reduction in survival with increasing dose of Mn(II)(3,5-DIPS)$_2$ would still hold.

The maximal and statistically significant ($P = 0.01$) increase in survival found for the 20 μmol $Cu(II)_2$(3,5-DIPS)$_4$ and 10 μmol Mn(II)(3,5-DIPS)$_2$ combination treatment supports the concept that treatment with a combination of metalloelement chelates provides a synergistic increase in survival of lethally irradiated mice. It may also be biologically significant that the ratio of Cu : Mn in this combination treatment is 2 : 1, 40 μmol Cu and 20 μmol Mn since the copper complex is binuclear and contains 2 atoms of copper while the manganese complex is mononuclear and contains 1 atom of manganese.

Acute toxicities for both the binulcear $Cu(II)_2$(3,5-DIPS)$_4$ and mononuclear Mn(II)(3,5-DIPS)$_2$ complexes have been reported to be 261 ± 36 μmol/kg or 91 ± 13 μmol/kg and 842 ± 92 or 706 ± 64 μmol/kg respectively for female or male C57BL/6 mice [1,2,9]. Both complexes have been shown to increase survival in $LD_{50}$ mice at a dose of 49 to 80 μmol/kg of body mass in female and male C57BL/6 mice when given subcutaneously or orally either up to 24 hrs before irradiation or up to 24 hrs after irradiation. The dose of irradiation for this experiment was selected to approach the $LD_{100}$ dose in order to more rigorously examine combination therapy with regard to its potential in facilitating survival of mice experiencing greater radiation injury. Results of these studies do support the possibility that combination therapy does offer the potential for recovery from increased radiation injury.

In this study the treatment and irradiation intervals ranged from three to eight hours starting with the control group and moving from left to right for the treatment groups shown in Table I and ending with the group treated with 40 μmol/kg $Cu(II)_2$(3,5-DIPS)$_4$ and Mn(II)(3,5-DIPS)$_2$/kg. This is not likely to have influenced the outcome of this experiment since, as we have shown [7,8], $^{67}$Cu administered as $^{67}$Cu labeled $Cu(II)_2$(3,5-DIPS)$_4$ was conserved and remained in blood, liver, kidney, intestine, muscle, lung, thymus, femur, spleen, and brain throughout the 5 day duration of our study. These complexes have also been found to increase survival of $LD_{50}$ irradiated mice when administered up to twenty-four hours before irradiation. Data presented in Table II do not reveal an influence of time of treatment before irradiation on survival. There were no statistically significant differences due to time of irradiation following treatment. However, this point remains to be addressed in future experiments when time before irradiation is held constant for all treatment groups.

Acknowledgements

We are indebted to the National Cancer Institute for a Summer Student Fellowship (V.A.P.), PHS grant number CA49425, the National Institute for General Medical Sciences, PHS grant number S06-RR08211 for a Student Fellowship (J.A.K.,III), the National Institutes of Health for two Research Apprenticeships (B.L.B. and T.B.), PHS grant number 5R25RR10281-03 and an Egyptian Government Scholarship (I.H.El-S.).

References

[1] J.R.J. Sorenson, L.S.F. Soderberg, L.W. Chang. *Proced. Soc. Exptl. Biol. Med.* **210**, (1995) 191.

[2] J.R.J. Sorenson, L.S.F. Soderberg, L.W. Chang, W.M. Willingham, M.L. Baker, J.B. Barnett, H. Salari, K. Bond. *European J. Med. Chem.* **28**, (1993) 221.

[3] T.D. Henderson, R.L. Burt, S.E. Kaufman, W.M. Willingham, J.R.J. Sorenson. *Radiat. Res.* **136**, (1993) 126..

[4] T.D. Henderson, R.D. Henderson, H.J. Irving, W.M. Willingham, J.R.J. Sorenson. *Inflammopharmacology* **3**, (1995) 241.

[5] L.S.F. Soderberg, J.B. Barnett, M.L. Baker, H. Salari, J.R.J. Sorenson. *Exptl. Hematol.* **16**, (1988) 577.

[6] L.S.F. Soderberg, J.B. Barnett, M.L. Baker, H. Salari, J.R.J. Sorenson. *Exptl. Hematol.* **18**, (1990) 801.

[7] C.G. Crispens, Jr., M.V. Chidambaram, D. Torregrosa, H. Salari, K. Bond, G.L. Kearns, R.A. Gray, C.E. Epperson, M.L. Baker, B. Griffey, S.J. Gilbreth, B.A. Garner, J.R.J. Sorenson. *Anticancer Res.* **9** (1989) 1213.

[8] J.R.J. Sorenson, L.S.F. Soderberg, M.L. Baker, J.B. Barnett, L.W. Chang, W.M. Willingham. Radiation Research Society meeting April 7-12, 1990.

[9] J.R.J. Sorenson, L.S.F. Soderberg, M.L. Baker, J.B. Baker, L.W. Chang, H. Salari, W.M. Willingham in "*Advances in Experimental Medicine and Biology, Volume 264, Antioxidants in Therapy and Preventive Medicine*", (L. Emerit, C. Auclair, and L. Packer. Eds.), .Plenum Press, New York (1990) 69.

**Received: March 15, 1999 - Accepted: March 31, 1999 - Received in revised camera-ready format: April 1, 1999**

